# Isolation, heterologous expression, and purification of a novel antifungal protein from *Bacillus subtilis* strain Z-14

**DOI:** 10.1186/s12934-020-01475-1

**Published:** 2020-11-23

**Authors:** Xuechao Zhang, Xiaojun Guo, Cuihong Wu, Chengcui Li, Dongdong Zhang, Baocheng Zhu

**Affiliations:** College of Life Science, Hebei Agricultural University, 289 Lingyusi Road, 071001 Baoding, PR China

**Keywords:** *Bacillus subtilis*, Antifungal protein, Purification, Heterologous expression, *Rhizoctonia cerealis*, Biological control

## Abstract

**Background:**

Wheat sheath blight, a soil borne fungal disease caused by *Rhizoctonia cerealis*, is considered as one of the most serious threats to wheat worldwide. *Bacillus subtilis* Z-14 was isolated from soil sampled from a wheat rhizosphere and was confirmed to have strong antifungal activity against *R. cerealis*.

**Results:**

An antifungal protein, termed F2, was isolated from the culture supernatant of Z-14 strain using precipitation with ammonium sulfate, anion exchange chromatography, and reverse phase chromatography. Purified F2 had a molecular mass of approximately 8 kDa, as assessed using sodium dodecyl sulfate polyacrylamide gel electrophoresis. Edman degradation was used to determine the amino acid sequence of the *N*-terminus, which was NH_2_ASGGTVGIYGANMRS. This sequence is identical to a hypothetical protein RBAM_004680 (YP_001420098.1) synthesized by *B. amyloliquefaciens* FZB42. The recombinant F2 protein (rF2) was heterologously expressed in the yeast host *Pichia pastoris*, purified using a Niaffinity column, and demonstrated significant antifungal activity against *R. cerealis*. The purified rF2 demonstrated broad spectrum antifungal activity against different varieties of fungi such as *Fusarium oxysporum*, *Verticillium dahliae*, *Bipolaris papendorfii*, and *Fusarium proliferatum*. rF2 was thermostable, retaining 91.5% of its activity when incubated for 30 min at 100 °C. Meanwhile, rF2 maintained its activity under treatment by proteinase K and trypsin and over a wide pH range from 5 to 10.

**Conclusions:**

A novel antifungal protein, F2, was purified from biocontrol *Bacillus subtilis* Z-14 strain fermentation supernatant and heterologously expressed in *Pichia pastoris* to verify its antifungal activity against *R. cerealis* and the validity of the gene encoding F2. Considering its significant antifungal activity and stable characteristics, protein F2 presents an alternative compound to resist fungal infections caused by *R. cerealis*.

## Background

Wheat (*Triticum aestivum* L.) sheath blight, a severe production constraint inducing heavy crop losses in epidemic regions, is found in almost all temperate wheat growing areas in the world and is a soil borne fungal disease caused by *Rhizoctonia cerealis* [1]. With the reform of planting systems, the popularization of high-yield varieties, and increases in water, fertilizer and density, the harm caused by wheat sheath blight has been become increasingly serious [2]. Hosts lack adequate resistance to wheat sheath blight disease, thought be the results of emerging virulent pathogen populations. Therefore, to manage the disease, alternative strategies, such as biological control, are required [3]. Among the species of bacteria with antifungal activity, *Bacillus* spp. are preferred because they produce endospores, making them very tolerant to desiccation and heat [4, 5]. *Bacillus subtilis*, a nonpathogenic and endophytic bacteria that exists widely in nature, has the ability to inhibit a variety of plant diseases [6]. *B. subtilis* is an important microbial population in agricultural soil and plant rhizospheres, which has broadspectrum antimicrobial activity and strong resistance to stress [7]. It produces many active substances such as enzymes, antibiotics, amino acids, and insecticides that have been used industrially and agriculturally [8].

During its growth, *B. subtilis* secretes a wide range of antimicrobial proteins to inhibit the growth of pathogenic microbes [9, 10]. Antimicrobial proteins have become an emerging research field because of their special mechanism of action, lack of serious environmental effects, and their infrequent induction of resistance in their target pathogenic species [11]. The biochemical characteristics and biological functions of purified antimicrobial proteins have been studied, and their effect on the histomorphology of the host or pathogen has been observed to clarify their site of action and antimicrobial mechanism [12]. After the physicochemical properties and antimicrobial activities of the purified proteins are determined, the genes encoding antimicrobial proteins can be obtained from the original bacteria [13]. The antimicrobial protein gene can then be transferred into the affected plant for expression, producing genetically engineered plants with disease resistance. Alternatively, the gene can also be introduced into a plant epiphyte or endophyte to construct high-efficiency biocontrol engineered bacteria [14].

Johnson et al. first reported that *B. subtilis* produced antimicrobial substances, and since then, many kinds of antimicrobial active substances have been found in different *Bacillus* strains [15]. Most antimicrobial substances produced by *B. subtilis* are low molecular peptides synthesized through the non-ribosomal pathway, including cyclic peptides, cyclic lipopeptides, and linear peptides, usually with a molecular weight of about 1000 Da [8]. However, there are also some protein antagonists synthesized via the ribosomal pathway. Identified antimicrobial peptides from *B. subtilis* can be divided into several groups, including bacteriocins, cell wall degrading enzymes (protease, chitinase, and glucanase), pathogenesis-related proteins, thaumatin-like proteins, non-specific lipid transfer proteins, and unknown proteins [13, 16, 17]. Bacisubin (molecular weight = 41.9 kDa), an antifungal protein isolated from *B. subtilis* B-916, has a strong inhibitory effect on a variety of pathogenic fungi. Bacisubin has ribonuclease and hemagglutination activity, but no protease or protease inhibitory activity [18]. The protein F3A, which was isolated from *B. subtilis* F3, has high homology with flagellin and shows good antimicrobial activity against *Monilinia fruticola* [19]. These proteins might be important to protect plants from pathogen infection. *B. subtilis* Z-14, selected from a wheat rhizosphere soil, demonstrated broad spectrum activity against phytopathogenic fungi in vivo and in vitro [20, 21]. The present study aimed to purify the novel antifungal protein secreted by strain Z-14, express it heterologously, and perform a preliminary characterization.

## Results

### **Purification of antifungal protein F2 from*Bacillus subtilis*strain Z-14**

**Fig. 1:**
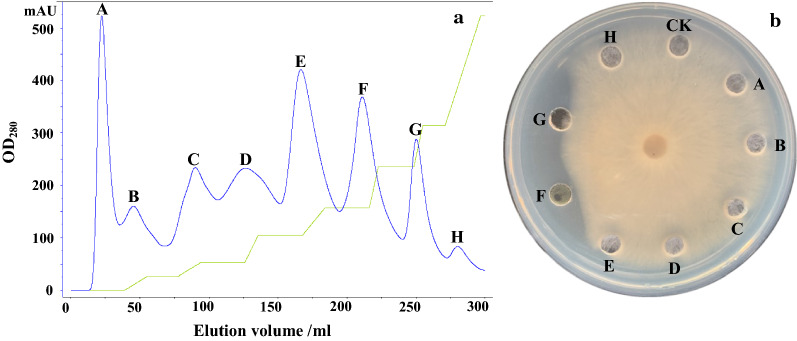
Chromatography to isolate an antifungal protein. Elution profile of the antifungal protein in the 80% ammonium sulphate precipitate separated using DEAE Sepharose Fast Flow ion-exchange chromatography (**a**). The antifungal activities of the separated proteins against the wheat pathogen *Rhizoctonia cerealis* (**b**). A–H represent separated fractions from the precipitate of the Z-14 strain fermentation supernatant [30 µg of each separated fraction dissolved in 30 µl of 10 mmol/l Tris–HCl buffer (pH 7.5)]; CK represents the negative control comprising 30 µl of 10 mmol/l Tris–HCl buffer (pH 7.5)

**Fig. 2:**
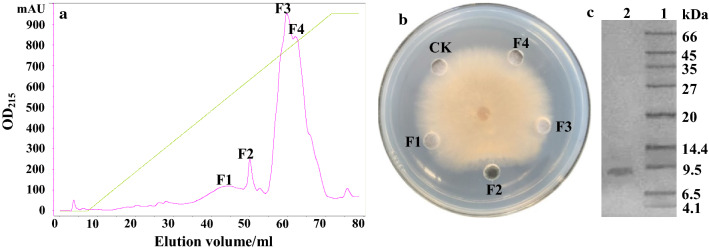
Purification of antifungal protein F2. Elution profile of fraction F obtained using reversed phase chromatography on a SOURCE™ 5RPC 4.6/150 column (**a**). The antifungal activities of the separated proteins against *Rhizoctonia cerealis* (**b**) and the purity and molecular mass of the purified antifungal protein F2 on SDS-PAGE (**c**). F1–F4 represent separated components from fraction F obtained using DEAE Sepharose Fast Flow ion-exchange chromatography [15 µg of each separated fraction dissolved in 30 µl of 10 mmol/l Tris–HCl buffer (pH 7.5)]; CK represents the negative control comprising 30 µl of 10 mmol/l Tris–HCl buffer (pH 7.5); lane 1: molecular weight marker; lane 2: the purified antifungal protein F2 from column chromatography

Anion-exchange chromatography was used to isolate antifungal proteins from crude extracts of strain Z-14 fermentation supernatant on a HiTrap diethylaminoethyl (DEAE)-sepharose fast Flow column, which resulted in unadsorbed fraction A and seven adsorbed fractions B–H (Fig. 1a). The antifungal activities of the eight fractions were determined using the test fungus *R. cerealis*. Only fraction F displayed a strong antifungal activity, with less antifungal activity being detected in fraction G (Fig. 1b). Reverse phase chromatography was used to further purify fraction F, which resulted in four main peaks (Fig. 2a). Among them, antifungal activity was only detected in fraction F2 (Fig. 2b) and sodium dodecyl sulfate polyacrylamide gel electrophoresis (SDS-PAGE) revealed a single band with a molecular mass of approximately 8 kDa (Fig. 2c). The protein was named as F2.

### **Amino acid sequence of the*N*-terminus of F2**

**Fig. 3:**
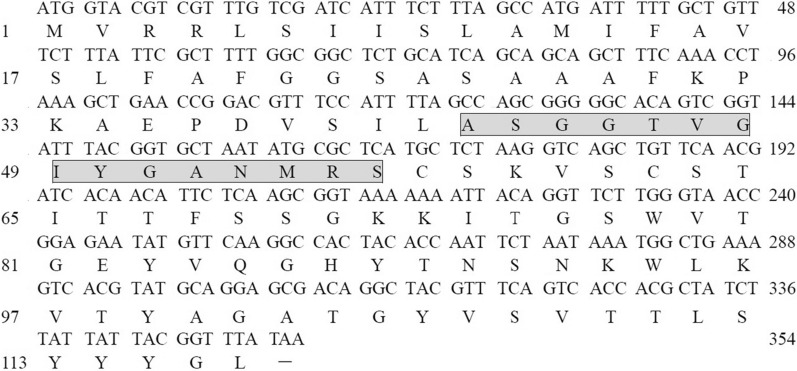
Nucleotide and the encoded protein sequence of the hypothetical protein RBAM_004680 (YP_001420098.1) derived from the genome of *B. amyloliquefaciens* FZB42 (NC_009725.1). Right side numbers refer to the nucleotide residues. Left side numbers refer to the encoded amino-acid residues. The gray rectangle indicates the 15 amino acids residues of antifungal protein F2 *N*-terminal segment from *Bacillus subtilis* Z-14 obtained by Edman degradation

The first 15 amino acids residues of the *N*-terminal segment of the antifungal protein obtained by Edman degradation were ASGGTVGIYGANMRS. After searching the National Center for Biotechnology Information (NCBI) protein database, we observed that the peptide was identical to that of a hypothetical protein RBAM_004680 (YP_001420098.1), which was derived from the genome of *B. amyloliquefaciens* FZB42 (NC_009725.1) [22]. Except for the amino acid sequence, no other information, such as its function, location, or mass has been reported. Taken together with our results, this indicated that F2 could be secreted by the bacteria, and acts on plant fungi in the environment. The gene encoding hypothetical protein RBAM_004680 has a 345 bp open reading frame (ORF) that encodes a protein of 117 amino acids with a theoretical molecular weight of 12.3 kDa and an isoelectric point of 9.635, as predicted by the EditSeq module of the bioinformatic analysis software LaserGene 8.1.3 (DNAStar, WI, USA) (Fig. 3). Using the gene sequence of the hypothetical protein RBAM_004680, a primer pair were designed and used to amplify the gene encoding antifungal protein F2 from *B. subtilis* Z-14. Sequence analysis of the polymerase chain reaction (PCR) product demonstrated that the F2 gene (*f2*) from strain Z-14 was identical to that of the hypothetical protein RBAM_004680 from strain FZB42. The 15 amino acids residues of F2 obtained by *N*-terminal sequencing were consistent with amino acids residues 42 to 56 of protein RBAM_004680. From the amino acid 42 to the end of RBAM_004680, the molecular weight was 8.04 kDa, which was consistent with that of protein F2. The antifungal protein F2 was isolated from the fermentation supernatant of strain Z-14, which implied that amino acids 1–41 of protein RBAM_004680 represent the signal peptide that allows F2 to be secreted into the extracellular space of strain Z-14, which was consistent with the result predicted by the SignalP software. We subjected the coding sequence and the antifungal function of the gene to the NCBI and acquired the accession number FJ225661. In the predicted three-level structure model of the hypothetical protein (or the antifungal protein), except for six beta folded stocks, the remaining structures are irregular curls. In addition, there are 33 hydrogen bonds and 10 corners, but no alpha helices were observed. The tertiary structure of the protein was a dense sphere, and most of the binding residues predicted by I-TASSER were located in a large groove of the sphere. The quality of the predicted structure of the protein was evaluated by SAVS and the results of the Ramachandran plot showed that 90.7% of the amino acid residues were in the allowed region, 5.3% in the general region and 4.0% in the disallowed region. The 3D–1D score of 76.92% amino acids was greater than 0.2.

### **Expression of the antifungal protein gene*f2*in*P. pastoris***

**Fig. 4:**
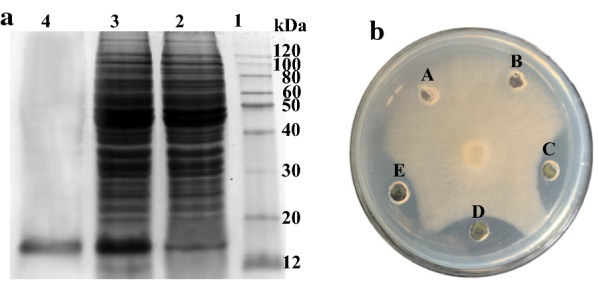
The purified recombinant protein rF2 has antifungal activity. SDS-PAGE analysis of the recombinant protein rF2 heterologously expressed in *Pichia pastoris* and purified using Niaffinity column (**a**). The antifungal activity of recombinant protein F2 (rF2) against *R. cerealis* (**b**). Lane 1: molecular weight marker; lane 2: total protein fraction from non-induced strain; lane 3: total protein fraction from the methanol-induced strain; lane 4: the recombinant protein rF2 purified by Niaffinity column; A, the negative control of 10 mmol/l Tris–HCl buffer (pH 7.5); B, the total fermentation supernatant proteins of *P. pastoris* before methanol induction; C, the total fermentation supernatant proteins of *P. pastoris* after methanol induction; D, the positive control of 10 µg of iturin A standard dissolved in 30 µl of 10 mmol/l Tris–HCl buffer (pH 7.5); E, 10 µg of the purified rF2 dissolved with 30 µl of 10 mmol/l Tris–HCl buffer (pH 7.5). An aliquot of 30 µl of each sample was added into the well, respectively

The antifungal protein gene *f2* was amplified using primers and successfully inserted into the plasmid pPIC9k. The recombinant plasmid was then transformed into *P. pastoris* GS115 competent cells. Positive colonies were identified by PCR and the subsequent antifungal activity was assessed. The fermentation supernatant of the recombinant strain exhibited strong antifungal activity against sheath blight pathogen *R. cerealis* and subsequent SDS-PAGE analysis demonstrated that a band around 13 kDa (corresponding to the theoretical molecular weight of rF2 and the added HIS tag) was only present in the fermentation supernatant of the recombinant strain; no such band was seen in the supernatant of the strain before methanol induction. Recombinant protein rF2 was purified using a Ni-affinity column from a culture induced for 96 h. After purification, a single band of approximately 13 kDa was observed in the eluate when analyzed by SDS-PAGE. Protein rF2 was eluted mostly in the 20 mmol/l imidazole fraction and almost no protein was found at higher concentrations of imidazole. The yield of purified rF2 was 65 µg/ml (Fig. 4a). Furthermore, the eluate samples were assessed for their antifungal activities and the obtained protein band was confirmed as recombinant protein rF2. rF2 demonstrated almost the same activity against *R. cerealis* as the iturin A standard, which implied that antifungal protein F2 possessed similarly strong antifungal activity to iturin A, which is a kind of lipopeptide antibiotic displaying strong broad spectrum antifungal activity (Fig. 4b).

### Antifungal activity of the recombinant protein against different fungi indicators

**Fig. 5:**
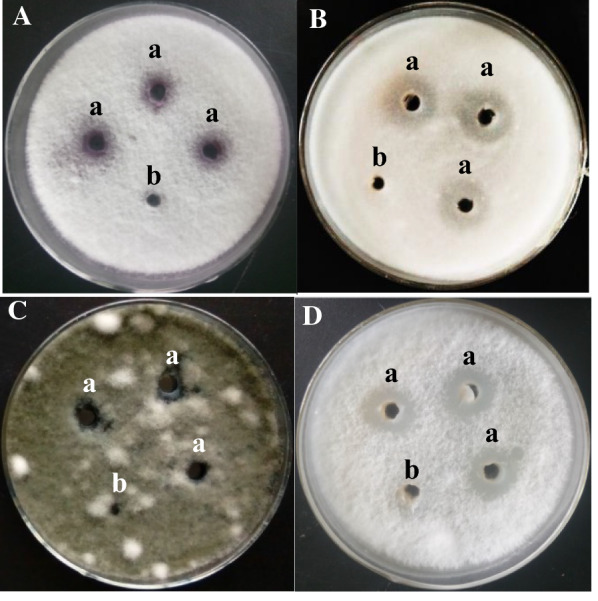
Antifungal activity of the isolated protein F2 excreted by *Bacillus subtilis* strain Z14 against different pathogenic fungi. **A** *Fusarium proliferatum*; **B** *Verticillium dahliae*; **C** *Bipolaris papendorfii*; **D** *Fusarium oxysporum*. ‘a’ Indicates that a 30 µl aliquot of rF2 (10 µg in total) was added and ‘b’ indicates that a 30 µl aliquot of 10 mmol/l Tris–HCl buffer (pH 7.5) was added as a negative control

The spectrum of the antifungal activity of the recombinant protein rF2 is shown in Fig. 5. rF2 presented a broad spectrum of antifungal activity against all four selected fungi indicators, with inhibition zones ranging from 0.84 cm (*Bipolaris papendorfii*) to 1.82 mm (*Fusarium oxysporum*) in diameter. rF2 demonstrated strong antifungal activity against *Fusarium oxysporum* and *Fusarium proliferatum*, but relatively weak antifungal activity against *Verticillium dahliae* and *Bipolaris papendorfii*. The activity of rF2 against *V. dahliae* displayed almost the same inhibition zone diameter as that of *F. oxysporum*; however, the inhibition zone was not as transparent as that of *F. oxysporum*, which implied that rF2 demonstrated higher antifungal activity against *F. oxysporum* than against *V. dahliae*.

### Effects of proteases, temperature, and pH on the activity of rF2

**Fig. 6:**
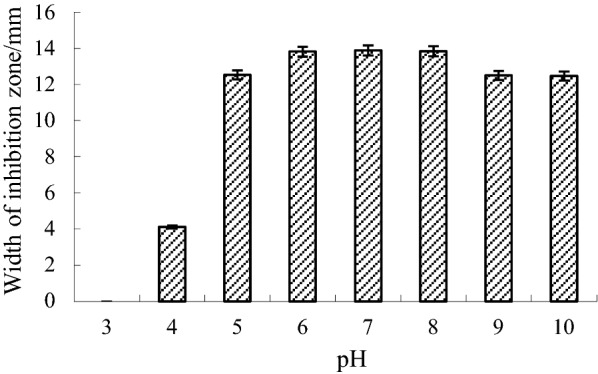
Analysis of the antifungal activity of recombinant protein rF2 under different pHs

The effect of pH on the antifungal activity of rF2 is shown in Fig. 6. The highest antifungal activity was detected at pH 7.0, which remained almost unchanged in the pH range 6–8. The activity decreased slightly at pH 5.0, 9.0, and 10.0, but decreased significantly, by 88%, at pH 4.0, and disappeared completely at pH less than 3.0. Protein rF2 preserved most of its antifungal activity after exposure to temperatures of 40–80 °C for 20 min, but lost 8.5% and 21.8% of its total activity when incubated for 30 min at 100 °C and 121 °C, respectively, which demonstrated that protein rF2 has strong thermal stability (Table 1). After pepsin treatment, rF2 lost 10.8% of its antifungal activity. However, the antifungal activity remained intact when treated with proteinase K and trypsin at 37 °C for 60 min (Table 2).

**Table 1:** Antifungal activity of recombinant protein rF2 treated under different temperatures

Temperature/°C	Diameter of inhibition zone/mm	Rate of activity loss (%)
CK	13.37 ± 0.12a	–
40	13.31 ± 0.11a	0.4
60	13.29 ± 0.18a	0.6
80	13.11 ± 0.16a	1.9
100	12.23 ± 0.15b	8.5
121	10.46 ± 0.10c	21.8

CK is the negative control comprising a 30 µl aliquot of 10 mmol/l Tris–HCl buffer (pH 7.5). The means within a column followed by different letters are significantly different from each other at* P* = 0.05

**Table 2:** Analysis of antifungal activity of recombinant protein rF2 treated with proteases

Proteases	Diameter of inhibition zone/mm	Rate of activity loss (%)
CK	13.57 ± 0.15a	–
Pepsin	12.10 ± 0.12b	10.8
Trypsin	13.41 ± 0.17a	1.2
Proteinase K	13.22 ± 0.12a	2.6

CK is the negative control comprising 30 µl aliquot of 10 mmol/l Tris–HCl buffer (pH 7.5). The means within a column followed by different letters are significantly different from each other at* P* = 0.05

## Discussion

The continuous use of chemical agents for biological control by crop growers, coupled with their lack of understanding of the mechanism of inhibition by these agents, has contributed to worldwide concerns regarding the use of chemical control of pathogens [23]. In addition, the successful use of a biocontrol agent depends upon being familiar with the biological environment in which the agent is to be used and the production of a stable formulation of the selected biocontrol agent. The use of antimicrobially active species and strains of *Bacillus*, or their metabolites, represents an alternative method of plant protection [24]. To support the use of known microorganisms or to develop of improved strains to control plant disease, it is necessary to understand the biological control mechanism at the biochemical and molecular levels, and to determine the underlying causes of the observed variability in biological control in agro-ecosystems [25]. The present study isolated and characterized a potential antifungal protein from *B. subtilis*. The protein is hypothesized to function in protecting wheat from infection by harmful pathogens. The antifungal activity of F2 could be used to resist sheath blight disease caused by *R. solani*, and thus could represent an effective biological control agent to efficiently manage the disease.

To date, few methods have been developed for large-scale protein separation. Extraction using ammonium sulfate precipitation coupled with column chromatography separation is still the main method for protein purification [14]. An antifungal protein from *B. licheniformis* HS10 of approximately 55 kDa was identified as a carboxypeptidase after isolation using 30–60% ammonium sulfate precipitation of culture supernatant combined with column chromatography purification on DEAE Sepharose Fast Flow, RESOURCE Q, and Sephadex G-75. The purified antifungal protein significantly inhibited eight kinds of plant pathogenic fungi, and showed stable biological activity after treatment for 30 min at 100 °C and at pH values ranging from 6 to 10 [26]. In the present study, an 8-kDa extracellular thermostable antifungal protein named F2 was demonstrated to have antifungal activity against plant-borne pathogenic fungi. F2 was purified using ammonium sulphate precipitation, anion exchange chromatography, and reverse phase chromatography, which demonstrated the column chromatography is still an effective technology for the separation of known or unknown proteins.

Antifungal proteins from *Bacillus* spp. have been used frequently to suppress various diseases. Over the last 10 years, researchers have identified several classes of proteins that inhibit fungal and bacterial growth using in vitro assays. These proteins are distributed widely in animals, plants, bacteria, and even in fungi, and might have vital functions in the protection of plants against infection by pathogens [11]. Using the 15 amino acids residues of the antifungal protein F2, the gene was cloned and submitted to NCBI for BLAST searching to identify 100 gene sequences with the closest homology. The result showed that the Max score ranged from 654 to 636, Query cover from 100 to 99%, E-value from 0.0 to 3e−178 and % identity from 100.00 to 99.15%. The homologous gene sequences all originated from *Bacillus* spp. such as *B. velezensis*, *B. amyloliquefaciens*, and *B. subtilis*, and were all obtained from genome sequencing. The functional annotations of these genes were scarce, with most of them being predicted to encode a hypothetical protein. The F2 gene product showed broad antifungal activity against many kinds of phytopathogenic fungi, which implied that F2 was a newly discovered antifungal protein. The identification of protein F2 enriched the antifungal protein family and has the potential to be used in agricultural production to control plant fungal diseases.

Structural analysis showed that the antifungal peptide rF2 was mainly composed of free coil and belonged to mixed type protein, which was consistent with a previous study that showed that the most secondary structure of an antimicrobial peptide was free coil [27]. The threedimensional structure of rF2 was compact, with the N-terminal and C-termini being very close. Rozek et al. showed that the activity of antimicrobial peptide indolicidin could be improved by bringing the two ends of the peptide close to each other [28]. Based on these analyses, it could be preliminarily inferred that the mechanism of action of antifungal peptide rF2 may be “carpet” mode, i.e., the peptide binds to the surface of target cell membrane, and when the concentration reaches a certain level, the cell membrane became permeable and forms many circular pores, which breaks the cell membrane and kills the target cells [29].

Unfortunately, the low production yield of these antifungal proteins from *Bacillus* spp. limits their practical application, despite our detailed understanding of their upstream transcription regulation elements that respond to stressors and environmental signals [30, 31]. For their future study and practical applications, it is necessary to produce antifungal proteins such as F2 in higher amounts in a non-sensitive, easily fermentable, ‘generally recognized as safe’ fungus. Several antimicrobial proteins have been used as the basis to design synthetic proteins and analogs as active ingredients of food preservatives, medicines, and commercial biopesticides [32]. The methylotrophic yeast *Pichia pastoris* is a popular expression system for the production of recombinant proteins at both laboratory research and industrial scale. *P. pastoris* has a high growth rate and the ability to grow to a high cell density on simple and inexpensive media. The expression of the recombinant *B. subtilis f2* gene was controlled by the strong and tightly regulated promoter of the alcohol oxidase (AOX1) gene, allowing the induction of gene expression using 0.5% methanol [33]. The coding sequence was also fused to the α-factor pro-peptide to ensure the secretion of the recombinant protein to the extracellular medium [32]. F2 has been expressed both in host *E. coli* and *B. subtilis*, but the yield and activity of the recombinant protein were negligible (data not shown). Recombinant F2 was expressed efficiently in *P. pastoris*, which could provide more economical materials for the application of antifungal proteins in agricultural production.

We successfully expressed protein rF2 in *P. pastoris* and used Ni affinity chromatography to purify it. This confirmed that the gene sequence correctly expressed antifungal protein F2. This efficient synthesis and simple purification will lay the foundation for followup applications. F2 is active over a wide pH range, and is resistant to high temperature and protease degradation, which is similar to the carboxypeptidase from *B. licheniformis* HS10 [26]. These characteristics, and the low costs of producing these antifungal proteins, identify them as promising candidates against pathogenic filamentous fungi.

In the present study, *R. cerealis* proved to be susceptible to protein F2 secreted from *B. subtilis* Z-14. Taking into account this susceptibility data and the characteristics of protein F2 (resistance against protease degradation, and stable antifungal activity over wide pH and temperature ranges); we believe that F2 represents an alternative compound to treat fungal infections caused by *R. cerealis*.

## Materials and methods

### Strains and culture conditions

*Bacillus subtilis* Z-14, which significantly reduced the growth of *Rhizoctonia cerealis* (a wheat sheath blight pathogen), was originally isolated from soil sampled from a wheat rhizosphere [20]. *Pichia pastoris* GS115 and pPIC9K were used as the host and vector, respectively, for heterologous expression of the recombinant antifungal protein F2. Z-14 was grown on nutritive agar (NA) overnight at 37 °C. An aliquot of the overnight culture was inoculated into 50 ml of fermentation medium (20 g of sucrose; 10 g of tryptone; 2 g of KH_2_PO_4_; 0.05 g of CaCl_2_; 0.05 g of MgSO_4_·7 H_2_O; and 1000 ml of distilled water; pH 7.5) in an Erlenmeyer flask and cultured for 48 h at 37 °C with shaking at 220 rpm.

### Antifungal protein extraction from the Z-14 supernatant

The Z-14 culture was centrifuged for 15 min at 10,000×*g* and the supernatant was retained and filtered through a 0.22-µm hydrophilic filter (Jinteng, Tianjin, China). The metabolites were precipitated from the supernatant using 80% saturated (w/v) (NH_4_)_2_SO_4_ and stored at 4 °C overnight. The mixture was centrifuged for 15 min at 10,000×*g* and the pellet was dissolved in 10 ml of 10 mmol/l Tris–HCl buffer (pH 7.5). To remove the ammonium sulfate, the solution was dialyzed in 2-kDa cut-off dialysis tubing (Sigma-Aldrich, St Louis, MO, USA) for 48 h at 4 °C, with a buffer change every 4 h (500 ml each). The dialysates were condensed using vacuum freeze-drying to yield precipitated proteins, which were further purified using column chromatography.

### **Antifungal activity assessment of the metabolites from Z-14 against*Rhizoctonia cerealis***

A diffusion plate assay method, modified from that described by Zhang et al. [34] was used to measure the antifungal activity of the metabolites from Z-14 strain. Evenly spaced wells (7 mm in diameter) were made 2.2 cm from the centre of potato dextrose agar (PDA) plates containing 40 µg/ml streptomycin sulphate. A plug (7 mm diameter) of *R. cerealis* fungus was placed in the centre of the plate and an aliquot (30 µl) of the metabolites or 10 mmol/l pH 7.5 Tris–HCl buffer (the control) was added to each well. The plates were incubated at 25 °C for 5 days and the inhibition zones were measured after clear halos became visible. The experiment was performed three times.

### Ion exchange chromatography purification of antifungal proteins

Ion exchange chromatography was carried out using a HiTrap DEAE-sepharose fast Flow column (Amersham Pharmacia, Uppsala, Sweden) equilibrated with 10 mmol/l Tris–HCl buffer (pH 7.5) on the ÄKTA explorer 100 system obtained from Amersham Biosciences (Sweden). The proteins bound to the column were eluted sequentially using 0.05, 0.1, 0.2, 0.3, 0.45, 0.6, and 1.0 mol/l NaCl. The eluate was assessed via its absorbance at 280 nm. Each fraction was dialyzed and adjusted to the same concentration using Tris–HCl buffer. The antifungal activity of each fraction was tested against *R. cerealis* using the agar-diffusion method described above.

### Purification of antifungal proteins using reverse phase chromatography

The fractions with antifungal activities were collected and subjected to reverse phase chromatography on a SOURCE™ 5RPC 4.6/150 column (Amersham Biosciences), which had been equilibrated using 0.06% trifluoroacetic acid (TFA). The material bound to the column was eluted linearly using 60% acetonitrile solution containing 0.05% TFA. Individual fractions were collected, subjected to dialysis, and then vacuum freezedrying was used to condense the fractions before further analysis.

### Purity, molecular mass, and concentration determination of the isolated proteins

SDS-PAGE was performed using 0.75-mm-thick gels comprising a 5% stacking gel and a 12% separating gel to assess the purity and molecular mass of the separated protein fractions. The Bradford method was used to determine the protein concentration, using bovine serum albumin as the standard [35].

### **Antifungal protein*N*-terminal sequence analysis**

The purified antifungal protein was separated using SDS-PAGE and then electroblotted onto a polyvinylidene fluoride membrane (Bio-Rad, USA) at 60 V for 30 min. The protein on the membrane was then applied to a 491 protein sequencer (Applied Biosystems, USA) to determine its N-terminal amino acid sequence using automated Edman degradation [36].

### **Construction of the expression plasmid pPIC9K-*f2***

The amino acid sequence homology of the *N*-terminus of the isolated protein was analyzed using the NCBI Basic Local Alignment Search Tool (BLAST) online search service to find similar proteins and related gene sequences. SignalP software (http://www.cbs.dtu.dk/serv-ices/SignalP/) was used to predict the signal peptide in the protein sequence. The 3D structure of the isolated antifungal protein was predicted using I-TASSER software (http://zhang.bioinformatics.ku.edu/I-TASSER/), and the quality of the predicted structure was evaluated by the structure prediction and evaluation software SAVS (http://nihserver.mbi.ucla.edu/SAVES/). According to the gene sequence and the multiple cloning sites of the expression vector pPIC9K, the primers W-QC: CCGGAATTCATGGTACGTCGTTTGTCGATC and W-D: ATAAGAATGCGGCCGCTTA GTGGTGGTGGTGGTGGTGTAAACCGTAATAATAAGATAG were designed, which incorporated EcoRI and NotI restriction sites in the PCR amplicon and added a sequence encoding a C-terminal His-tag. The primers were used to PCR amplify the target gene encoding the antifungal protein using Z-14 genomic DNA as the template. The PCR amplicon, encoding a His-tagged protein named *f2*, was ligated into expression vector pPIC9K via the EcoRI and NotI sites. The ligation products were transformed into *Escherichia coli* DH5α cells. Positive transformants were selected and screened for presence of the recombinant plasmid. The recombinant plasmid was validated using DNA sequencing.

### **Recombinant protein expression in*Pichia pastoris*GS115**

SacI was used to linearize the expression plasmid pPIC9K-*f2*, which was then transformed into electrocompetent *P. pastoris* GS115 cells using a model 165–2100 MicroPulser Electroporator (Bio-Rad, Hercules, CA, USA) following the manufacturer’s instructions. The obtained transformant culture was spread on plates comprising yeast potato dextrose (YPD) agar medium containing 2 mg/ml G418. Single colonies that appeared after incubation at 25 °C for 2 days were picked out and detected using PCR with primers W-QC and W-D. For recombinant protein production, a positive transformant was inoculated into buffered glycerol-complex medium (BMGY) and cultured at 25 °C for 24 h, with shaking at 250 rpm. Centrifugation was used to harvest the cells, which were resuspended in 50 ml of buffered methanol-complex medium (BMMY) in a 500 ml flask. Recombinant F2 (rF2) expression was induced for 96 h and methanol was added every 24 h at 1% (v/v) final concentration. The fermentation broth was centrifuged at 10,000×*g* and 4 °C for 10 min, and the supernatant was collected to measure the antifungal activity and for SDS-PAGE. Iturin A standard (Sigma–Aldrich) was used as the positive control to compare the antifungal activities against *R. cerealis* with that of rF2.

### Purification of the recombinant antifungal protein

A His60 Ni Superflow™ Resin & Gravity Column (Clontech Laboratories, Mountain View, CA, USA) was used to purify the HIS-tagged recombinant protein following the manufacturer’s protocol. Bound proteins were eluted successively using 20, 50, 100, and 200 mmol/l imidazole. The eluate was dialyzed to remove the imidazole and condensed using vacuum freeze-drying. The precipitate was dissolved in 10 mmol/l Tris–HCl buffer (pH 7.5) and the solution was used to detect the antifungal activity and for SDS-PAGE. The antifungal activity of the purified recombinant protein against *R. cerealis* was tested using the agar-diffusion method as described above.

### Assessing the antifungal spectrum of the recombinant protein rF2

Four fungi: *F. proliferatum*, *V. dahliae*, *B. papendorfii*, and *F. oxysporum*, were selected as indicators to assess the antifungal range of rF2. The antifungal activities of rF2 against the fungal indicators were tested using the agar-diffusion method [34]. Fungi were cultured on PDA slants at 25 °C for 7 d. Water was then added to the slants, the surface mycelia of which were rubbed gently with a glass rod to harvest the conidia. The fungal spore suspension (10 ml; 1 × 10^6^/ml) was mixed with 100 ml of PDA and poured into Petri dishes (n = 6). After the PDA solidified, agar disks were excised to form wells with a diameter of 7 mm, into which were added 30 µl-aliquots (10 µg in total) of rF2 solution. The same volume of 10 mmol/l Tris–HCl buffer (pH 7.5) served as the control. For each sample, three replicates were performed. The plates were incubated for 5 days at 25 °C. The antifungal activity was determined as the diameter of the growth inhibition zone around the wells compared with that of the control well.

### The influence of proteases, temperature, and pH on the activity of recombinant protein rF2

To analyze the effect of different pHs (range 3.0–10.0) on the antifungal activity of rF2, the pH of the culture filtrate was altered using 2 mol/l NaOH or HCl and incubated for 2 h at 37 °C. Protein rF2 was exposed to a range of temperatures (40, 60, 80, 100, and 121 °C) for 30 min to study its thermal stability. To detect its stability under protease treatment, rF2 was digested using 1 mg/ml protease K, trypsin, and pepsin (Amresco, Radnor, PA, USA) for 60 min at 37 °C, respectively. The antifungal activities of the rF2 culture filtrates treated as detailed above were determined according to the method described by Zhao et al. [37]. The formula reported by Wong et al. was used to calculate the relative activity of the treated rF2 protein [38]. Three replicates for each treatment were assessed in three repeated experiments.

### Statistical analysis

Replicate data are expressed as the mean ± the standard deviation (SD). The Statistical Product and Service Solutions (SPSS) ver. 17.0 software package (IBM Corp., Armonk, NY, USA) was used to perform all the statistical analyses. One-way analysis of variance and Duncan’s test (*P* ≤ 0.05) were used to assess whether the means differed significantly.

## Abbreviations

*R. cerealis*: *Rhizoctonia cerealis*
*B. subtilis*: *Bacillus subtilis*
*B. amyloliquefaciens*: *Bacillus amyloliquefaciens*
SDS-PAGE: Sodium dodecyl sulfate polyacrylamide gel electrophoresis
DEAE: Diethylaminoethyl
NCBI: The National Center for Biotechnology Information
ORF: Open reading frame
*P. pastoris*: *Pichia pastoris*
PCR: Polymerase chain reaction
PR: Pathogenesis-related
NA: Nutritive agar
PDA: Potato dextrose agar
BLAST: Basic Local Alignment Search Tool
YPD: Yeast potato dextrose
BMGY: Buffered glycerol-complex medium
BMMY: Buffered methanol-complex medium
SPSS: The Statistical Product and Service Solutions
